# Recent Progress of Fluorescence Sensors for Histamine in Foods

**DOI:** 10.3390/bios12030161

**Published:** 2022-03-04

**Authors:** Gan Wu, Xilin Dou, Dapeng Li, Shihan Xu, Jicheng Zhang, Zhaoyang Ding, Jing Xie

**Affiliations:** 1 College of Food Science and Technology, Shanghai Ocean University, Shanghai 201306, China; m210300839@st.shou.edu.cn (G.W.); m200300830@st.shou.edu.cn (X.D.); dpli@shou.edu.cn (D.L.); 2 Department of Chemistry, University of Washington, Seattle, WA 98195, USA; xushihan@uw.edu (S.X.); jicheng@uw.edu (J.Z.)

**Keywords:** fluorescence sensors, histamine, quantum dots, aptamers, food safety

## Abstract

Biological amines are organic nitrogen compounds that can be produced by the decomposition of spoiled food. As an important biological amine, histamine has played an important role in food safety. Many methods have been used to detect histamine in foods. Compared with traditional analysis methods, fluorescence sensors as an adaptable detection tool for histamine in foods have the advantages of low cost, convenience, less operation, high sensitivity, and good visibility. In terms of food safety, fluorescence sensors have shown great utilization potential. In this review, we will introduce the applications and development of fluorescence sensors in food safety based on various types of materials. The performance and effectiveness of the fluorescence sensors are discussed in detail regarding their structure, luminescence mechanism, and recognition mechanism. This review may contribute to the exploration of the application of fluorescence sensors in food-related work.

## 1. Introduction

Biological amines are produced when aldehydes and ketones are transaminated and amino acids are decarboxylated as basic nitrogen compounds [1]. They are low-molecular-weight organic bases synthesized by the metabolism of microorganisms, vegetables, and animals [2]. An increase in biogenic amine concentration indicates food spoilage and poses a threat to food safety. Histamine has received much attention for its lack of dietary safety and negative effects on the human body. Not all bacteria have amino acid decarboxylases, but Cocci and Streptococcus can decarboxylate one or more amino acids [3,4,5]. Some microorganisms can produce a lot of histamine, such as Morganella (Proteus), Klebsiella (Klebsiella pneumae) strains and Aluya (Aluea) strains. These microorganisms are of high value in the process of microbial research in fish products [6,7,8]. The histamine concentration of ground beef inoculated with Proteus morgani was 595 pg/g, and that of ground beef inoculated without Proteus morgani was 8.26 pg/g [9]. Histamine is also produced by glowing bacteria, which are halophilic, and it is reported that there are intermediate and colophilic species. Pseudomonas has also been identified as a histamine-producing bacterium in fish [8]. Bifidobacterium (Bifidobacterium botulinum, Clostridium botulinum, respectively), Lactobacillus brucei, Lactobacillus cucurbita, Lactobacillus botulinum, and Lactobacillus hielii isolated from meat products are all amine-producing lactic acid bacteria [10,11]. Additives are used in the production of fermented foods and beverages, in which the starter can potentially affect the production of biogenic amines by affecting the interaction between microbial populations. However, these processes can cause great trouble when they occur in food spoilage.

Although histamine is essential for many key functions in humans and animals, eating foods with high levels histamine may have unhealthy effects. There have been many reported errant foodborne poisonings caused by histamines. Several cases of histamine poisoning have occurred after intaking corrupt cheese or fish [12,13]. The toxic effects of histamine are due to the interaction of two receptors (H and Hz) on human or other biological cell membranes that come into contact with the histamine. The effects of histamine toxicity mainly manifest in peripheral vascular, capillary, and arterial dilation, and further manifest as hypotension, skin flushing, and headache [14]. The abdominal cramps, diarrhea, and vomiting that occur during this process may result from the H-receptor-mediated gastric histamine-induced contraction of intestinal smooth muscle [15]. Histamine regulates gastric acid production via Hertzian receptors in parietal cells, which may explain the symptoms found in some cases of food poisoning caused by histamines [16]. Of the biogenic amines that can be detected in foods such as seafood, cheese, alcoholic beverages and meat products, histamine is currently probably the most toxic [17]. The toxicological effects of histamine depend on the concentrations ingested by the individual during histamine intoxication, as well as other amines, ammonia oxidase activity, and individual intestinal physiology. There is a fairly efficient detoxification system in the mammalian gut that metabolizes biogenic amines consumed in a normal diet. Under normal circumstances, exogenous amines absorbed by the body from food can be rapidly detoxified by amine oxidase or coupling reactions, but not in allergic individuals or those with monoamine oxidase inhibitors. Amino-oxidase is an inducing enzyme in the presence of monoamine or diamine [18]. In the process of detoxification, monoamine oxidase and diamine oxidase have had an impact that cannot be ignored. However, some enhancers (classified as food and putrefactive amines or pharmacological agents) inhibit the activity of these enzymes, thus limiting the detoxification process. Thus, histamine in rotten fish or over-fermented cheese is more toxic than histamine in a water solution due to the presence of these substances. In addition, histamine-metabolizing enzymes are also inhibited by certain antihistamines, antimalarial drugs, and other drugs [19].

The Association of Official Analytical Chemists (AOAC) procedure is the method used by official U.S. testing agencies to detect histamine in food [20]. This detection method obtains information on histamine content by detecting the fluorescence intensity in the processed sample. The sample processing process includes methanol homogenization, filtration, loading to an anion exchange column, and finally, derivatization by phthalaldehyde. Furthermore, various fluorescence-based techniques have been reported for the detection of histamine in fish. In addition to these methods, some chromatographic methods are also used for histamine detection. However, there are few reports about the simultaneous detection of different types of bioamines.

**Scheme 1:**
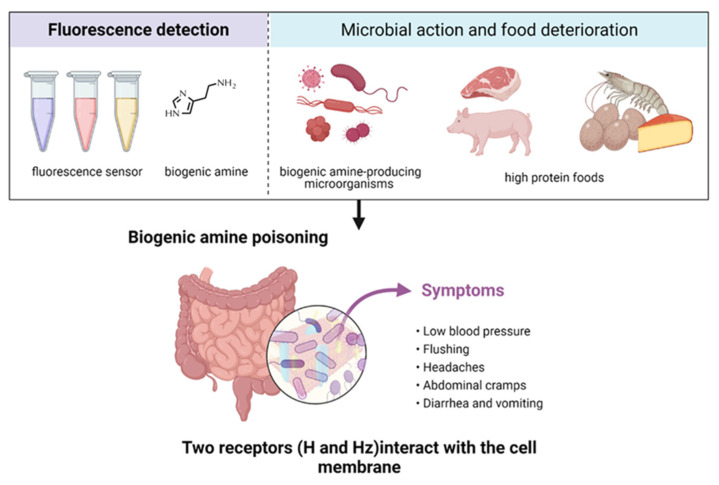
Biogenic amine poisoning and fluorescence sensor detection of histamine.

This paper mainly introduces the application and development of fluorescence analysis for histamine detection in food, which is of great significance to food safety (Scheme 1). Because of the diversity and adaptability of histamine fluorescence detection, we introduce fluorescence sensors from the following aspects with diverse cases. First of all, for fluorescence sensors, their main materials, chemical molecular structure design and addition of functional groups determine the functions and characteristics of the sensor. Then the synthesis process of the fluorescence sensors and their application in food detection are introduced, in the context of foods such as tuna, trout, and related food cans and other raw food materials, food products and semi-finished products. These contents can reflect the operability and simplicity of the sensor well. Finally, we summarize the fluorescence sensor, a food safety detection tool with great potential, and predict its future development.

## 2. Histamine Detection

Histidine decarboxylase can convert histidine to histamine by virtue of its catalytic activity. At the same time, histidine decarboxylase is mainly composed of an imidazole ring and an aliphatic amino group connected by a chain of two carbon atoms, all of which are basic, which can be protonated in acidic solution [21,22]. The effects of histamine activity on human functions mainly include allergy, digestion, and neurological function, but can also extend to other processes related to wound healing, circulatory diseases, immunology, oncology, and infectious diseases [23]. Histamine poisoning occurs when the content of histamine exceeds the minimum safe intake for humans. Fish containing >50 mg of histamine in 100 g of fish can cause histamine poisoning in fish. In order to ensure the error tolerance rate, food regulations in the United States have clarified that the histamine content in 100 g of fish should not exceed 5 mg, while Europe requires that the histamine content in 100 g of fish does not exceed 10 mg [24,25]. Although histamine is rapidly inactivated by diamine oxidase in healthy individuals, even increased concentrations of histamine in the blood can cause severe symptoms [26]. In addition to the main symptoms that cause IgE-mediated food allergies, the ingestion of histamine-rich foods, alcohol, or drugs that release histamine or conduction block diamine oxidase may cause diarrhea; hypotension; arrhythmia; bronchoconstriction and nasal conjunctiva inflammation; urticaria; or headache. These may confuse the diagnosis of food allergy [27].

Histamine poisoning was first recorded in 1828 and was recorded as a food-borne disease caused by eating spoiled fish [28]. In the most recent case of malignant poisoning, the deceased died from food poisoning due to high histamine content in boiled crabs. In this case, it is worth noting that histamine exhibits high-temperature resistance and is easily dissolved in water [29], which means that histamine is a potential food safety hazard that is heat resistant and not easily observed.

### 2.1. Conventional Histamine Detection Techniques

Much of the early widespread use of histamine detection methods relied on colorimetric assays, using eye (or visual colorimeter) observation, comparing the color depth of solutions, or measurement with a photoelectric colorimeter to determine the amount of histamine in a solution. For amine concentration, colorimetric analytical methods have been improved, for example, by overcoming the reaction of calcium in bone with potassium hydroxide (potassium hydroxide) to adjust pH during experiments [30]. There is also a simple extraction of histamine, further interaction with imidazole, and detection of histamine based on quantitative chromogenic reagents and reference color standards [31]. Furthermore, the use of 2,3-naphthalenedicarbaldehyde (NDA) can also be used as a chromogenic reagent for the detection of histamine [32]. It needs to be purified in many colorimetric detection methods, or used together with other methods in a form similar to dual response [33]. It has good performance in the detection of a limited histamine concentration range. At the same time, there are also improved methods for the combined application of fluorescence sensors and colorimetric analysis methods, which broaden the detection range of colorimetric analysis methods and reduce the detection limit [34]. The use of the fluorescence sensor alone can also realize the visual detection of colorimetric analysis methods.

At the same time, many detection methods today also rely on chromatographic separation techniques. Thin-layer chromatography (TLC), gas chromatography, high-performance liquid chromatography (HPLC) and ultra-high-performance liquid chromatography (UHPLC) cooperate with various detectors to detect histamine in food. Histamine was detected in [35,36,37,38]. However, most of these detection methods are accompanied by a lot of time and consumption while obtaining ideal detection data. With few exceptions, most of the histamine analysis methods are based on the early technology of TLC or the powerful HPLC developed in recent years, which requires the support of laboratories with sophisticated equipment to obtain relatively accurate but limited number of samples. Combining these separation methods with fluorescence sensors can undoubtedly improve complex detection environments. However, fluorescent sensors can gain selectivity through special reaction processes or functional groups to overcome complex detection environments. It is precisely because fluorescence sensors can be modified to obtain better performance, in addition to their own series of material advantages, that they are favored by many researchers. The following detailed description of the sensor effectively reflects the above point of view.

### 2.2. Quantum Dots as Fluorescence Sensors

Quantum dots (QDs) are also called semiconductor nanocrystals, generally smaller than 10nm in size, and are clusters composed of a small number of atoms or molecules [39]. Current literature reports mainly concern the II–V groups, such as CdSe; I–V subgroups, such as InP, InAs, and GaAs compounds; and elements such as Si [40]. The structure of QDs endows a size-quantum effect and dielectric confinement effect, thereby obtaining unique luminescent characteristics [41]. The use of luminescent QDs as fluorescent markers in immune reactions is a new field of fluorescence immunoassay used in recent years [42,43]. Most QDs currently used in immunoassays use CdSe, CdS, or ZnS as the core [44,45,46], which is a shell-core-shell nanocrystal. Compared with the above-mentioned fluorescent probe, the nanocrystal has the advantages of high-fluorescence quantum yield, high photochemical stability, and minimal photobleaching after multiple excitations. Therefore, laser-induced fluorescence can be used. Excitation and emission spectra can be controlled by changing the size and composition of the nanoparticles. The Stokes shift is large, and the fluorescence emission peak is narrow and symmetric (generally, the half-value width is 20~30 nm). The excitation spectrum has a wide range of wavelength, and the same excitation source can be detected several times at the same time. As the diameter of the nanocrystal decreases, the emission wavelength shifts to a shorter wavelength. Lastly, the marking technique is very simple and can be widely used in food safety testing [43].

A large proportion of polymers are hydrophobic materials synthesized from organic macromolecules. The methods of making quantum dots include the microemulsion method and reprecipitation method. In the microemulsion method [47], the polymer material is first dissolved in a non-polar organic solvent; then, the dissolved polymer is added to pure water in which a surfactant is dissolved. The solution is then shaken in ultrasonic waves, at which point the polymer solution becomes a stable and dispersed microemulsion droplet. Then, the organic solvent is removed, and the polymer nano-aqueous solution with stable monodisperse particle size in the range of 30–270 nm can be obtained. In the reprecipitation method [48], the polymer is first dissolved in an organic solvent (such as tetrahydrofuran THF); then, the polymer organic solution is rapidly injected into pure water during the violent vibration of ultrasonic waves. The interaction of the two heterogeneous solvents is the polymer aggregation, and sedimentation occurs to form an organic nano-mixed solution. Finally, the organic solvent is removed to obtain a uniformly dispersed and stable polymer nano-aqueous solution. Since no surfactant is required in the reprecipitation method, the deficiency of the surfactant to the fluorescent nanoparticles can be avoided. Since the size of the nanoparticles prepared by the reprecipitation method is affected by the initial solution concentration of the polymer, the higher the concentration, the larger the particle size; furthermore, the particle size of the synthesized nanoparticles can be regulated by controlling the water content of the initial solution. Moreover, this method is simple in operation, low in cost and high in yield, so it is widely used in the preparation of quantum dots.

#### 2.2.1. Molecular Imprinting Quantum Dots

A new type of fluorescence sensor was synthesized for the determination of histamine dihydrochloride by modifying a molecularly imprinted polymer (MIP) layer on carbon nanoparticles (CNPs) prepared from tragacanth gum [49]. Initially, CNPs were prepared in one step by hydrothermal synthesis from a tragacanth gum aqueous solution. Then, the MIP layer was fixed on the surface of CNP through a sol-gel process to form MIP-coated CNPs (MIP-CNPs). The MIP was prepared by the reverse microemulsion method, the template (histamine dihydrochloride) was mixed with the imprinting material for a period of time to fully polymerize, and then the stability of the reverse microemulsion was destroyed by acetone. The template was separated by centrifugation and solvent, and finally, the MIP was obtained. The excited state charges tended to return to the original orbital and lead to quenching of fluorescence, which was manifested in the transfer of charges between the conduction band of the CNPs and the lowest unoccupied molecular orbital (LUMO) of the template molecule. The fluorescence intensity of MIP-CNPs decreased linearly with the increase in histamine dihydrochloride concentration in the range of 6.2 nM–0.17 μM, with a detection limit of 1.5 nM. In the experiment of Behzad Rezaei et al, the hydrothermal treatment of tragacanth was used to prepare carbon dots (CDs).

Another type of fluorescence sensor based on molecularly imprinted polymer manufactured through an organogel process is described in [50]. The sensor composition includes molecularly imprinted nanofibers as receptors and CdSe/ZnS quantum dots as fluorescence sensors. First, quantum dots and histamine templates were added to the polymerizable gelling agent for organogelation, and through gel state polymerization and template removal, QD-doped histamine-imprinted organogel nanofibers (QD-HIOGNF) were finally obtained. Acrylates with carboxyl groups were used as functional monomers complexed with templates in this process. With the help of a photoinitiator and cross-linking agent, the gel was polymerized, and highly cross-linked organogel nanofibers were obtained by means of ultraviolet irradiation. The template was detached from the sensor by dissolution with a methanol/acetic acid (9:1 *v*/*v*) solution. The obtained QD-HIOGNF presented high polymer recognition properties in terms of sensitivity and selectivity to histamine. The fluorescence intensity of QD-HIOGNF was sensitively quenched with increasing histamine concentration. Meanwhile, QD-HIOGNF can be reused for sensing after the removal of bound analytes.

#### 2.2.2. Ionic Liquid Quantum Dots

**Figure 1:**
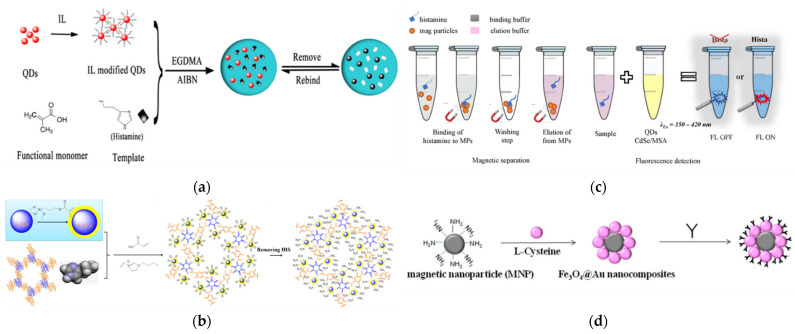
(**a**) Schematic diagram of the preparation of QDs@IL@MIP [53]: (**b**) schematic diagram of the synthesis of a stable covalent-organic-framework fluorescent probe using ionic liquids and S-doped carbon dots [54]; (**c**) CdSe Quantum dots/MSA detection process [63]; (**d**) preparation of Fe3O4@Au magnetic nanocomposites [67].

Ionic liquids can improve the fluorescence stability of QDs, and can also provide quantum dots with C=C double bonds to better bind to the target molecules [51,52]. Qing-Hua Wang et al. [53] used a simple one-pot method in CdSe/nS QDs. The sensor material was synthesized on the surface of the dots. The ionic liquid (IL) was successfully introduced through electrostatic interaction to obtain QDs@IL@MIP (Figure 1a). Amine groups were bonded to the surface of the quantum dots through N-C bonding, which effectively eliminated the surface defects of the quantum dot, and therefore increased the fluorescence emission intensity. Finally, the practicality of the sensor was verified by the histamine detection of canned trout, canned tuna, canned sardine, and canned saury.

#### 2.2.3. Element-Doped Quantum Dots

The CD fluorescence sensor was improved by using S-doped ionic liquid to embed the sensor into metal–organic framework (MOF) organic analogs composed of organic units through strong covalent bonds (covalent organic frameworks, COF) (Figure 1b), thus obtaining sulfur-doped carbon dots (S-CDs) [54]. Due to electron transfer, histamine statically quenches fluorescent probes and can be analyzed using the Stern–Volmer equation. Because the excellent performance of COF can improve the performance of the fluorescence sensor—and S-doped ionic liquid can improve the fluorescence intensity, quantum yield, and life span—the fluorescence stability and sensitivity of S-CDs can be improved to a certain extent compared with undoped CD. Finally, S-CDs were applied to the detection of histamine in red wine, yellow rice wine, white wine, canned fish, fermented pork, and chicken sausage, and the detection results also effectively verified the above views. In another example, a common method was used to prepare COF-derived N-CDs@MIP in a reversed microemulsion system of Triton X-100 and cyclohexane, by using silane reagents, to functionalize one-pot polymerization [55]. Likewise, doping nitrogen into carbon-based fluorescent sensing systems can also enhance the optical performance. This is because nitrogen-doped carbon dots (N-CDs) can tune the work function by inducing charge delocalization, and enhance the electron transfer ability in carbon-based materials, resulting in a larger visible light absorption region.

A highly fluorescent N-CD was prepared using a one-step melting method of urea and glycine [56]. In order to determine the appropriate Ag^+^ concentration, 10 μM, 30 μM and 50 μM Ag^+^ were selected for the synthesis of quantum dots and tested at an excitation wavelength of 370 nm. Through the quenching effect of Ag^+^ on the fluorescence of N-CDs, as well as the change in fluorescence intensity caused by the competitive binding of N-CDs and biogenic amines to Ag^+^, the histamine molecule also has its unique fluorescence response in this process. During the use of N-CD@Ag^+^, biogenic amines with a minimum differentiation concentration of 500 nM allowed researchers to perform excellent qualitative analysis.

#### 2.2.4. Graphene Quantum Dot

Graphene quantum dots (GQDs) were also used as fluorescence sensors [57]. To investigate the photoluminescence of histamine in aqueous dispersions of amino-functionalized graphene quantum dots (GQDs-amino, with an average size of 28 nm), various metal ions were introduced. The results showed that Eu^3+^, Fe^3+^ and Cu^2+^ could promote the recognition of histamine by GQDs-amino. The data show that the presence of Fe^3+^ can improve the recognition ability of GQDs surface up to 10 times. The linear response of GQDs-amino-Fe^3+^ (345/435 nm) to histamine concentration ranged from 0.43 μM (LOD) to 32 μM. Meanwhile, GQDs-amino-Fe^3+^ was also applied to the analysis of histamine in tuna samples after cationic solid phase extraction.

#### 2.2.5. Functional Group Quantum Dots

In the following detection method, compared with conventional FIA (enzyme-linked amplified fluorescence immunoassay), the sensitivity can generally be improved by two orders of magnitude. Ashish Yadav et al. [58] used β-cyclodextrin (β-CD) embedded in zinc oxide QDs and modified with vitamin B6 cofactors, pyridoxal 5′-phosphate (PLP) and pyridoxal (Py), to obtain β-CD embedded and PLP/ Py modified host-guest complexes. Histamine was detected using the prepared ZnO@PLP and ZnO@Py. When histamine was added, selective fluorescence enhancement at 473 nm and 460 nm could be achieved. Under ideal conditions, the detection capabilities were 2.49~24.4 μM and 7.44~47.6 μM, respectively. The detection limits were 0.59 μM and 0.97 μM, respectively. Similarly, weak luminescent silver nanoparticles (AgNPs) decorated with β-CD (β-CD-AgNPs) with a size of about 7 nm were synthesized and interacted with 5′-pyridoxal phosphate [59]. The vitamin B6 cofactor PLP resulted in a transient fluorescence enhancement at 532 nm, due to the inclusion and complexation of PLP with the interaction of β-CD modified on AgNPs.

Water solubility is also a factor that cannot be ignored in the biological application of quantum dots. Biocompatibility can be achieved by a polymer ligand system that provides water-soluble and effective anchoring groups. We can polymerize and functionalize quantum dots through ligand exchange. For example, stable fluorescent particles with a high quantum yield were prepared, and the histamine functional polymer with a poly(ethylene glycol) side chain was prepared by RAFT polymerization [60]. A multifunctional post-modification strategy of activated ester units of NMS (N-methacryloxysuccinimide) and poly methacrylate (ethylene glycol) was employed in the polymer chains, providing a low polydispersity copolymer of 6 K to 50 K, with a customized composition of each monomer along the copolymer chain. The effect of PEGMA molecular weight on yield is relatively stable (about 30%) during the production process, and the lower polymerization temperature seems to be suitable for the production of most quantum dots. At the same time, it is necessary to control the time and monomer ratio in the research of quantum dot functionalization. Ultimately, it is effectively used for histamine detection.

CdTe quantum dots modified with thioglycolic acid (TGA-CdTe QD) have been studied for histamine detection [61]. The synthesis was performed using methods reported in the literature [62]. The obtained dispersion of thioglycolic-acid-modified CdTe QD in water was used as a probe. The interaction between TGA-CdTe QD appears to be electrostatic quenching in nature. Under optimal conditions, the analysis response followed the Stern–Volmer model. In the experiments, satisfactory recovery rates were obtained in the detection of canned and fresh tuna samples.

Histamine could be separated by magnetic particles and quantified by changes in the fluorescence intensity of CdSe quantum dots modified with Mercaptosuccinic acid (MSA) [63]. After Fe_2_O_3_ particles were formed, TiO_2_ was adsorbed on the surface. When mixed with magnetically separated histamine, the fluorescence intensity of CdSe QD increased with increasing concentration; the limit of detection (Figure 1c) and the limit of quantitation were 1.6 µM and 6µM, respectively.

Phage display technology can quickly screen peptides. The selected peptides can selectively bind histamine with high affinity and specificity, thereby enhancing the selectivity and sensitivity of the QDs [64,65]. Through covering the surface of carbon quantum dots with N-acetyl-l-cysteine (NAC-CQDs), NAC-CQDs with high stability and photoluminescence were obtained [66]. Moreover, the peptide specifically bound to histamine, obtained by the phage display method, can quench the fluorescence of NAC-CQDs. After adding histamine, the hydrophobic domain of histamine and the target peptide will cause the peptide to escape from NAC-CQD and restore fluorescence. Therefore, the recovery of fluorescence intensity can indirectly quantify the content of histamine. The detection range of histamine under this detection system is 0.1–100 ppm, with a detection limit of 13.0 ppb.

#### 2.2.6. Gold Nanoparticles

CD was bound to the silanized nanoporous alumina membrane using glutaraldehyde as a crosslinker [67]. The citric acid reduction method was used to produce AuNPs with a size of 10 nm. Fe_3_O_4_@Au nanocomposites were obtained by mixing a certain ratio of AuNPs and L-cysteine-modified MNPs (10:1) at 4 °C and leaving for 12 hours (Figure 1d). The CD immobilized on the nanoporous alumina film served as a donor and provided a fluorescent emission structure for histamine detection. Fe_3_O_4_@Au magnet nanocomposites concentrate histamine in fish samples as receptors. The Fe_3_O_4_@Au magnet nanocomposite material quenches the fluorescence signal of CDs and gives feedback on the histamine concentration through FRET mechanism. Histamine have been separated from fresh mackerel and storage mackerel and detected using a fluorescence quantitative analysis system. AuNPs have also been applied for fluorescence measurement of DNA containing the oxidative damage product 8-hydroxy-2′-deoxyguanosine (DNA-8-OHdG) [68]. Through the specific immune reaction between 8-OHdG and the antibodies on AuNPs, AuNPs bound tightly to CDs.

In another case, a fluorescence sensing platform was developed based on the CQDs@ZIF-8@Apt-AuNPs probe [69]. It is worth noting that AuNPs (fluorescence quenchers) have both quenching and enhancing effects on CQDs@ZIF-8: quenching fluorescence at 490 nm and enhancing fluorescence at 657 nm. This phenomenon can be explained by FRET and IFE theories. Among them, ZIF-8 can act as the anchor point and signal modulator of CQDs to modulate the fluorescence changes of CQDs through the ICT effect. This sensor is characterized by simplicity and high sensitivity.

### 2.3. Metallic Material as Fluorescence Sensors

Some rare earth metal ions (such as Eu^3+^, Tb^+^, Sm^3+^, etc.) can form strong fluorescent complexes with some ligands (such as β-diketone derivatives, polyamine carboxylic acids, etc.). This type of complex is absorbed by the ligand, and the energy is transferred from its excited singlet state (S) to the resonance energy level of the rare-earth-metal ion through its triplet state (T) and emits fluorescence in a narrow band [70,71,72]. The light absorption region of the ligand is generally 250~360 nm, and its Stokes shift is about 250 nm. The fluorescence lifetime of rare-earth-metal-ion complexes is about 50~1000 μs, which is 103~106 times longer than the background fluorescence lifetime of biological samples. Therefore, the interference of background fluorescence can be completely overcome using time-resolved techniques, which can greatly improve the measurement sensitivity of fluorescence sensors [73].

#### 2.3.1. Metal–Organic Framework

Hybrid porous materials with different functions, namely MOFs, can be fabricated by the combination of different organic ligands and metal ions [74]. Some double-/multiple-emission MOFs recognize amines through electron-delocalization-induced emission redshift, limit-induced enhancement, and energy transfer [75,76,77]. However, MOF-based BA ratio luminescent sensors have rarely been implemented [78,79,80]. The dual-/multi-emission MOF fluorescence response behavior of biogenic amines is difficult to control, because residual fluorescence, energy transfer between different donors and recipients, and structural stability all affect the scaling of MOFs. Therefore, in some complex environments, the detected concentration range often exceeds the actual concentration. The countermeasure taken by most detection modes is a scaled measurement with a fixed reference signal. Moreover, the fluorescence signal change generated when the fluorescence sensor probe is exposed to biogenic amines does not change rapidly within the required detection range, so it is limited when integrated with a portable sensor terminal and used for on-site detection.

**Figure 2:**
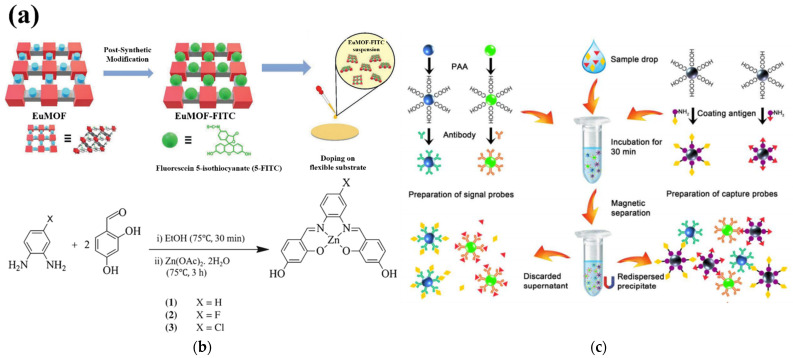
(**a**) Schematic diagrams of the preparation of EuMOF-FITC and EuMOF-FITC loaded films [81]; (**b**) synthesis of zinc (II) salphen complexes [86]; (**c**) schematic diagram of material preparation for multiple fluorescence immunoassays [95].

A new sensor used Intelligent Assessment System MOFs based on the fluorescent metal–organic matter to visually monitor the freshness of food [81]. Through post-synthetic modification, fluorescein 5-isothiocyanate was modified by covalent coupling with NH_2_-rich lanthanide MOFs, resulting in ratiometric fluorescent MOF probes (Figure 2a). The obtained ratiometric fluorescent probe can achieve a dual-emission response to histamine: on the one hand, the fluorescence intensity of fluorescein isothiocyanate (FITC) increases, and on the other hand, the fluorescence intensity of Eu^3+^ decreases, resulting in a clearly distinguishable fluorescence color shift from orange–red to green. By adding probes onto flexible substrates, the resulting MOF composite films can be applied for smart portable platform integration. In another case, MOF shows a novel two-fold 2D→2D parallel entanglement method. The two-dimensional Cd(II) MOF is constructed from tris(pyridine)-based hexacarboxylate zwitterionic ligands [82]. It is this entanglement that determines the close interlayer contact between the carboxylate (electron donor) and pyridinium (acceptor) which, in turn, endows the MOF with reversible photochromic properties through photo-induced electron transfer. This is an extension of photo-induced-electron-transfer-based photochromism from bispyridinium to polypyridinium compounds. Due to the light response behavior, the fluorescence of MOF can be reversibly modulated or switched by light irradiation.

A new strategy proposed in the design of another MOF sensor is a new application of molecular logic systems in the field of sensing [83]. Methyl red@lanthanide metal organic frameworks (MR@EuMOFs) were produced by covalently modifying MR into NH_2_-rich EuMOFs, which have a high quantum yield (48%). Through the energy transfer from the ligand to Eu^3+^ and MR, a dual stimulus–response fluorescence center was generated. By dispersing and solidifying MR@EuMOFs in the aqueous sodium salt of carboxymethyl cellulose, a portable sensory hydrogel was obtained. The hydrogel exhibits a color shift when its “smells” histamine vapor. This shift of the emission peak based on MR was closely related to the histamine vapor concentration. A rational analytical device was constructed with one-to-two logic gates, in which the concentration of histamine vapor was the input signal and the emission intensity of the two fluorescences was the output signal.

In a reported work, we found that metalloporphyrins containing Cu^2+^ and Zn^2+^ can bind tightly to histamine, although the binding strength of metalloporphyrins to histamine is not entirely dependent on the metal center [84]. The stability of six metalloporphyrins, including (Cu^2+^ or Zn^2+^)-benzoate, tosylate, and carboxylates with different peripheral metals, was investigated by exposing the compound and histamine to different temperatures. In some cases, the side chain of porphyrin can strongly affect the stability of the complex. This possibility means that the system has a special selectivity for histamine.

Used as a fluorescent chemical sensor for detecting histamine, the nanofiber PCL-Por and PCL-Por (Zn) mixed with dendritic porphyrin were manufactured by electrospinning [85]. The electrospun nanofibers produced by this method have interconnected pore structures and narrow pore size distribution. When the histamine solution was contacted with PCL-Por (Zn) nanofibers, a changed fluorescence response spectrum was produced. Fluorescence intensity decreases with increasing histamine concentration, which also applies to lower concentrations of histamine solution. Histamine acts as a quencher in this process, because Zn in Den-Por (Zn) comes into contact with histamine to form a complex, which is then introduced into PCL-Por (Zn) fibers by coordination.

The first attempt at complete optimization was made, in order to make an optical sensor based on metal salphen and use it to detect histamine in food [86]. In this case, Salphen, N, N'-phenylene bis(salicylene) is a Schiff base, which can be prepared by the condensation reaction of one equivalent of diamine and two equivalents of salicylaldehyde (Figure 2b). The combination of zinc (II) complexes without electron-withdrawing groups (complex 1), and zinc (II) complexes with electron-withdrawing groups (F, complex 2; Cl, complex 3), with histamine, will result in enhanced fluorescence signals. Since complex 2 has better optical properties than complex 1 and complex 3, complex 2 was selected as the sensing material of the bio-amine photosensor. Using silica particles as a fixed carrier, the histamine in the shrimp samples was analyzed to test the recovery performance of the sensor.

In the design of porphyrin-based sensors, using a rarely used b-functionalization strategy, amine accepting sites were attached to the framework of zinc (II) porphyrin molecules to provide a two-position chemical sensor [87]. The b-pyrrole functionalization strategy was used to construct a two-position chemical sensor for binding histamine. The supramolecular binding interaction between host and guest was firmly established by combining spectroscopic methods and computational analysis. The unique features of this chemical sensor design may open up a way to complement current developments in supramolecular porphyrin chemistry.

The Co(II) ion complex of a cyanine dye interacts with histamine and produces a rapid and intense change in fluorescence intensity. This fluorescence sensor is fabricated on the basis of changing the position of the ligand [88]. In the absence of contact with histamine, the complexed heavy metal ions (M^2+^) have a quenching effect on the fluorescence of the probe, and the fluorescence of the probe is not clear. When the probe is in contact with histamine, the metal in the probe will be dissociated in a competitive binding manner, thereby showing a strong change in fluorescence intensity. Although some Ni(II) complexes can also detect histamine under the same principle, their probes have a slower signal response to histamine and lower sensor reproducibility than Co(II).

#### 2.3.2. Organic–Inorganic Hybrid Nanomaterials

One review introduces the material concept, synthesis, and characterization of inorganic–organic hybrid nanoparticles (IOH-NPs) [89]. IOH-NPs are a new type of fluorescence detection and optical imaging nanomaterial with many excellent properties: high fluorescent dye loading (70–85 wt%), good photochemical stability, direct hydration, simple material, high emission intensity, easy absorption by cells, and safety. In addition to their full-emission applications, IOH-NPs can also be used for multimodal imaging, singlet oxygen generation, and drug delivery and release. Calcein and perylene tetra carboxylate will quench their fluorescence after forming hybrid nanoparticles. When they are in contact with histamine, together with fluorescent dyes, they will show strong fluorescence. The fluorescence intensity is related to the amount of histamine added. In further research, four new types of IOH-NPs have been proposed, which can detect histamine by fluorescence [90]. These IOH-NPs are Cu^2+^ [Calc]^2−^; Ag^2+^ [Calc]^2−^ (Calc: Calcein, C_30_H_24_N_2_O_13_); Cu_2_^2+^ [PTC]^4−^; and Ag^4+^ [PTC]^4−^ (PTC: perylene tetracarboxylic acid ester, C_24_H_8_O_8_). They contain extremely high calcein dye loading (90 wt% in Cu^2+^ [Calc]^2−^), 70 wt% in Ag^2+^ [Calc]^2−^) and perylene derivatives (Cu_2_^2+^ [PTC]^4−^ in 77 wt%, Ag^4+^ [PTC]^4−^ in 50 wt%). The salt-water compound was water-based to provide a highly stable nanoparticle suspension. Fluorescence from calcein and perylenetetracarboxylic acid was quenched by solid nanoparticles. When histamine was added, the fluorescence emission intensity increased with the increase in histamine concentration. Among them, Ag^+4^ [PTC]^4−^ showed the best performance (the addition of 100 µM histamine enhanced 180 times).

#### 2.3.3. Fluorescent Copper Nanoparticles

Highly photoluminescent self-assembled copper nanoclusters (Cu NCs) can quickly, sensitively, and selectively detect histamine [91]. By using 2,3,5,6-tetrafluorothiophenol as a reducing agent and protective ligand, Cu NCs were synthesized under simple conditions. Self-assembly-induced emission exhibited a strong saffron yellow color (590 nm). The absolute quantum yield of highly photoluminescent assembly was as high as 43.0%. The sensor system was used to analyze the histamine content in fish, shrimp, and red wine. Luminescent test strips based on Cu NCs were manufactured for the colorimetric detection of histamine in food.

A fluorescence sensor based on the aggregation-induced emission (AIE) effect [92], D-penicillamine-terminated copper nanoparticles (DPA-CuNPs), was synthesized by a one-pot method on the basis of copper nanoparticles, in which D-penicillamine was a reducing agent and stabilizing ligand [93]. Due to the AIE effect, DPA-CuNPs can generate a relatively strong red fluorescence signal (650 nm). When DPA-CuNPs came into contact with histamine, they dispersed into uniform nanoparticles and exhibited fluorescence quenching. The fluorescence sensor fabricated based on the above principles had a linear detection range of histamine from 0.05 μM to 5 μM with a detection limit of 30 nM.

#### 2.3.4. Upconversion Luminescence

A dual-mode probe for the rapid extraction and sensitive detection of histamine can be synthesized by modifying MIPs doped with AgNPs onto the surface of upconversion particles (UCNPs) [94]. When the probe was contacted with histamine, the fluorescence intensity of UCNPs@MIPs-AgNPs gradually weakened, while the SERS intensity gradually increased. Meanwhile, the fluorescence mode showed lower detection limits, and quantification limits of 81 nM and 0.18 μM.

In the fluorescence sensor developed by Biao Zhang et al. [95], the antihistamine antibody was connected with NaYF4Yb and Er (550 nm emission) UCNP to form a polychromatic signal probe. The histamine-coating antigens were attached to magnetic microspheres as capture probes. The fluorescence signals of competing immune complexes formed at 550 nm represented the concentrations of histamine (Figure 2c). The linear range of the immunoassay was 4.5 nM–0.9 μM, and the limit of detection of histamine was 0.9 nM–0.9 μM.

In a report, Ga^3+^ and Lu^3+^ doping were used to improve the upconversion photoluminescence of GdAlO_3_: Er^3+^ and Yb^3+^ phosphors [96]. GdAlO_3_:Er^3+^ and Yb^3+^ phosphors doped with Lu^3+^ and Ga^3+^ ions were prepared by a co-precipitation method. The effect of Ga^3+^ replacing Al^3+^, and Lu^3+^ replacing Gd^3+^, on the phosphor structure and upconversion photoluminescence (UCPL) was studied. The experimental results showed that the crystal structure was not changed by Lu^3+^ and Ga^3+^ doping, but the lattice parameters awerere slightly changed. This led to a reduction in the energy of the host phonon and a significant improvement in the 546 nm green emission spectrum. In addition, the amount of surface CO_2_, CO_3_^2−^ and OH^−^ species gradually decreased with the doping of Lu^3+^ and Ga^3+^. This combined effect led to the improved efficiency of Ga^3+^ and Lu^3+^ doped UCPL.

### 2.4. Aptamer Fluorescence Sensors

The most commonly used method of early histamine detection is to obtain a highly fluorescent adduct by condensing it with o-phthalaldehyde after organic extraction [97]. A typical extraction method is bis(2-ethylhexyl)phosphoric acid (B2EHPA) ion pair reagent, which can be used to highly selectively extract histamine from various biological fluids and tissues of humans and rats [98]. This method is more specific than n-butanol to extract histamine and is not affected by ranitidine. However, the efficiency of this detection method, which requires a complicated extraction process, is not ideal. The application of nucleic acid aptamers solves these problems in the fluorescence detection of histamine.

**Figure 3:**
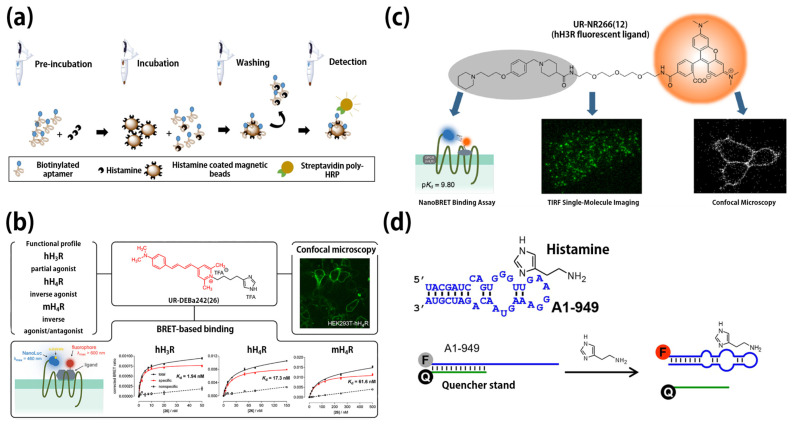
(**a**) Schematic representation of the detection of histamine using a competitive assay on beads. Reprinted with permission from [99]. Copyright^© 2022^, American Chemical Society; (**b**) Py-5-labeled UR-Deba242 multipurpose fluorescent probe for detection of histamine H_3_ and H_4_ receptors. Reprinted with permission from [103]. Copyright^© 2022^, American Chemical Society; (**c**) standard H_3_R ligands were labeled with UR-NR266. Reprinted with permission from [104]. Copyright^© 2022^, American Chemical Society; (**d**) the histamine aptamer A1-949 (sequence and secondary structure mimicked by Mfold) was combined with a fluorescent label (a blue label bound to the 5' end) for histamine detection. Binding of the DNA strand as a quencher to the histamine aptamer resulted in fluorescence quenching. Competitive binding of histamine and aptamer resulted in the recovery of fluorescence [105].

AThe classic systematic evolution of ligands by an exponential enrichment (SELEX) process design used magnetic beads for target fixation, and was completed after ten rounds of selection [99]. The sequences obtained from the previous round of magnetic bead screening were subjected to next-generation sequencing, and the appropriate sequences were preliminarily screened by aptamer PCR affinity analysis. The identification of H2 aptamers was based on the structure-function and characterization of the candidates. The affinities of the H2 aptamer and histamine were validated with four independent assays (KD of 3–34 nM). The obtained H2 aptamer was applied to magnetic bead separation to competitively identify histamine content in buffer and synthetic urine (Figure 3a), yielding detection limits of 18 pM and 76 pM in both solutions, respectively, while no matrix effects were observed.

The aptamer binding affinity was determined by a magnetic bead-based enzyme-linked oligonucleotide assay; then, unmodified histamine was detected by electrochemical impedance spectroscopy (EIS) at physiological pH. Aptamer H47 (GCCTGTTGTGAGCCTCCTAACATTTCTATGCTGCAGCCAACTTTTCCATACTTCCAGCTTACCATTTATCCATGCTTATTCTTGTCTCCC) showed the lowest apparent binding affinity (72.8 ± 13.9 nM) to bead-fixed histamine [100]. When immobilized on a gold surface, H47 showed the largest biosensor response in the presence of dissolved histamine compared with other single-stranded DNA sequences (ΔRct = 6.83 ± 2.00). Compared with other similar small molecules, the H47 EIS aptamer sensor also showed a highly selective and concentration-dependent response to histamine (linear range = 1 µM−5 mM). The H47 EIS aptamer sensor had an obvious binding affinity, detection limit, and quantification limit of 7.80 ± 1.70 mM, 4.83 mM, and 16.08 mM, respectively, which supports the prospect of developing aptamer sensors in applications for the quick detection of histamine in solution

#### 2.4.1. Aptamer Binds to Fluorescent Peptide

An excellent functional histamine fluorescent RNP sensor was developed by custom screening of RNA subunits as ligand-binding regions [101]. During the production process, the N-terminus of the peptide of the Rev peptide motif was labeled with a fluorescent substance to obtain the ability of a signal transducer. Selective RNP fluorescence sensors with better optical properties and binding properties could be screened by RNP pools consisting of RNA subunits and Rev peptide motifs. When the histamine and the fluorescent RNP sensor (Complexes of RNA aptamers and fluorescent Rev peptides) were combined, the fluorescence intensity changed obviously, thus revealing the detection process of histamine. The H05/7mC-Rev (H05 UGGGAAUAACAAUAGU CCUAACUGGCAACU) used in the sensor fabrication showed good selectivity to histamine.

#### 2.4.2. Aptamer Binds to Gold Nanoparticles

Based on salt-induced particle aggregation with the participation of histamine—the specific recognition of histamine molecules by aptamers and gold nanoparticles—a gold nanoparticle aptamer fluorescence sensor was fabricated, wherein when histamine comes into contact with the sensor, it shows a fluorescence change from red to blue [102]. The AuNP size, salt type and concentration, and aptamer concentration were optimized in the experiments, resulting in a detection limit of 8 nM for histamine. The aptamer used in the sensor was the H2 histamine aptamer (5′-AGC TCC AGA AGA TAA ATT ACA GGG AAC GTG TTG GTT GCG GTT CTT CCG ATC TGC TGT GTT CTC TAT CTG TGC CAT GCA ACT AGG ATA CTA TGA CCC CGG-3′).

#### 2.4.3. Aptamer-Binding Small-Molecule Fluorescent Probe

For histamine H_3_ receptors (H_3_R), especially H_4_R homologs (for example, human (h) and mouse (m)), there is a great need for fully characterized fluorescent probes as a multifunctional tool to supplement radioligands. In view of the use of fluorescent probes for BRET-based binding studies and H_4_R localization in living cells, Py-5 labeled histamine derivatives were synthesized (Figure 3b) [103]. UR-DEBa242, acting as a partial agonist at the hH_3_R, had a function as an inverse agonist or antagonist at the h/mH_4_Rs.

UR-NR266 (12), as a submolar-affinity fluorescent ligand, had good selectivity in the detection of histamine receptors [104]. UR-NR266 (12), labeled with 5-TAMRA, showed good kinetic and fluorescence characteristics, and had good adaptability to NanoBRET detection (Figure 3c). Due to its specific binding and high fluorescent brightness, UR-NR266 (12) can be clearly observed under fluorescence microscopy and is the first fluorescent ligand to image an H_3_R single molecule. In addition, it enables the acquisition of single-molecule images in saturated concentration imaging buffers, and compensates for potential fluorophore photobleaching.

#### 2.4.4. Aptamer Binds to Fluorescent Dye

Recently, an aptamer fluorescence sensor was developed based on an RNA aptamer (A1-949 aptamer) with a specific recognition ability for histamine through a structure switching mechanism [105]. The 5′ end of the aptamer A1-949 was modified with a fluorescent label, and the 3′ end of the aptamer was hybridized with a DNA strand, with a quenching function complementary to the aptamer. At this time, the fluorescence was in a quenched state. When histamine contacted the aptamer fluorescence sensor, the quenching DNA was displaced and released to restore the fluorescence of the sensor (Figure 3d). Enantiomeric versions of the aptamer sensors (L-RNA and L-DNA) were synthesized after optimization, which enabled sensitive and more robust detection of achiral analytes. Finally, fish samples with known histamine concentrations were tested and compared with the assay results of commercially available enzyme assay kits to verify the effectiveness of the aptamer fluorescence sensor (BioAssay Systems, Hayward, CA, USA, EnzyChrom Histamine Assay Kit).

### 2.5. Organic Small Molecules and Organic Polymers as Fluorescence Sensors

#### 2.5.1. Organic Small Molecules

The selective and rapid detection of histamine can be carried out by the combined application of capillary electrophoresis and light-induced fluorescence detection technology [106]. This assay used 4-Fluoro-7-nitro-2,1,3-benzoxadiazole (NBD-F) as a fluorescent derivatizing reagent to label histamine, and the NBD-F-labeled biogenic amines were rapidly separated in phosphate buffer solution (85 mM) at pH = 3. The method showed good performance in the determination of histamine in tobacco mesophyll protoplast lysate. Further, since this method can selectively analyze biological samples, it can be used as a potential method to study the effect of biogenic amines on the calluses and embryos of protoplast cultures.

A simple synthetic fluorescein dye exhibited good fluorescence properties [107], and was further used to conduct dual-mode fluorescence detection of histamine [108]. The detection principle was to achieve a specific response to histamine through the imine formation reaction and secondary hydrogen bond reaction catalyzed by imidazole, while maintaining pH = 7, during the detection of histamine in water (human serum and urine). While it rapidly identified histamine, it is noteworthy that other neurotransmitters or cellular metabolites did not appear to cause changes in the spectral signature of the luminescent dye. The quenching of fluorescein by histamine during the detection process showed static quenching. At the same time, the fluorescein is also used in portable color detection strips and fluorescence imaging detection of histamine in cells. The fluorescein dye in this method overcame the problems of biological toxicity and complex interferences of some types of fluorescence sensors, to a certain extent.

**Figure 4:**
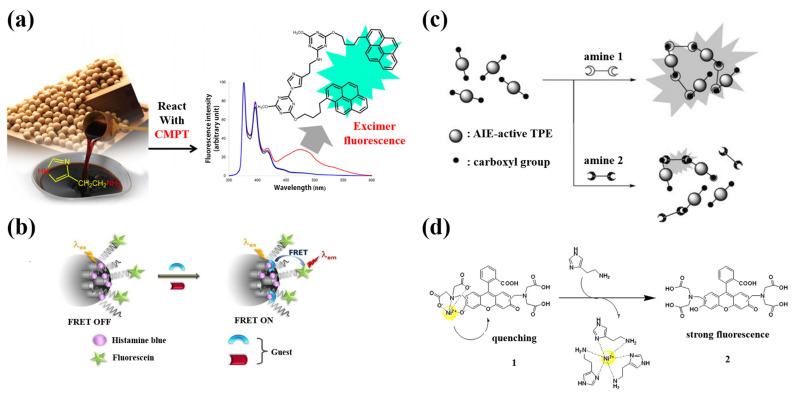
(**a**) An excellent excimer fluorescence derivatization reagent, 2-chloro-4-methoxy-6-(4-(pyren-4-yl)butoxy)-1,3,5-triazine, for the analysis of histamine in Japanese soy sauce by a highly sensitive and selective liquid chromatography method [109]; (**b**) schematic of a fluorescence sensor that can selectively detect Him or His [120]; (**c**) schematic diagram of TPE-based biogenic amine fluorescence sensor [121]; (**d**) the sensing mechanism of histamine using complex between Ni^2+^ and calcein [122].

A new type of pre-column excimer fluorescence derivatization reagent was developed for histamine detection [109]. During the derivatization of 2-chloro-4-methoxy-6-(4-(pyrene-4-yl) butoxy)-1,3,5-triazine (CMPT), histamine was converted to a polypyrene derivative, and the fluorescence of intramolecular excimers was released at 475 nm. At the same time, the control reagent emitted normal fluorescence at 375 nm to distinguish it from histamine derivatives. Compared with traditional excimer fluorescent derivatization reagents, CMPT has better chemical stability and can maintain reaction stability in solution for at least 36 days. The fluorescence sensor was successfully applied to the sensitive and selective analysis of histamine in different kinds of commercial Japanese soy sauce (Figure 4a).

A two-part fluorescent probe, Nile Red, and iminodiacetic acid-Ni^2+^ complex were used to detect histamine in cells [110]. The probe detection strategy for histamine was based on the ligand exchange reaction between histamine and iminodiacetic acid on the M^2+^ ion in the probe. When the M^2+^ ion in the probe was coordinated with the histamine molecule, it dissociated from the probe, and the fluorescence characteristics of the probe were restored. To maximize the quenching efficiency of M^2+^ ions, a Mannich reaction was used to synthesize the probe. In the composition of the probe, the iminodiacetic acid (IDA)-M^2+^ complex served as the hydrophilic part, and the Nile red dye served as the hydrophobic part. The probe had good adaptability in the detection of histamine in various cells and organs, and at the same time, did not require auxiliary reagents to increase the permeability of cell membranes or to carry out relevant modifications to cells in advance.

Primary amines can be converted to the corresponding aldehydes and ammonia using methylamine dehydrogenase (MADH). MADH was isolated from P. versutus grown on minimal medium, and 150 nM MADH was added to 20 mM HEPES buffer pH 7.5 and reacted for 6 minutes in the experiment. The reducing equivalents produced in the above process were transferred to amicyanin, the physiological chaperone of MADH. Amicyanin converted from blue (oxidized state) to colorless (reduced state). The change of absorption could be reflected by the change in the fluorescence of the Cy5 dye covalently modified on the sensor. The dye was limited, quenched by the Cu-site of the amicyanin due to fluorescence resonance energy transfer (FRET) [111]. The quenching efficiency and labeled fluorescence in this process were mainly affected by the oxidation state of amicyanin [112]. By observing the ratio between the concentration change of histamine and the change of fluorescence intensity, the concentration of histamine added to MADH/amicyanin-Cy5 could be detected with high sensitivity.

The fluorescent substance concentrated in the liposome microcapsules was self-quenched and the fluorescence was very weak after being released in the solution. It showed strong fluorescence due to the dilution effect, so it could exert a very effective amplification effect [113,114,115]. Vivek K. Bajpai et al. [116] derivatized antihistamine IgG with a maleimide functional group (the combination of maleimide-derived antihistamine IgG and sulforhodamine B-encapsulated liposomal nanovesicles, after removing the acetylthioacetate group from sulforhodamine B-encapsulated liposomal nanovesicles). Finally, the rupture mechanism of thiohodamine B dye-coated antihistamine antibody-coupled liposome nanobubbles was used for liposome-based histamine determination. The content of histamine in fresh mackerel, canned tuna and salmon, red meat powder, and ready-to-eat salad samples was detected by the method of standard recovery.

The BODIPY group is a dye with excellent fluorescent properties, and also a high-affinity hH_3_R ligand. In one study, bodilisant, a sensor with strong green fluorescence, was created by coupling the histamine H_3_ receptor (hH_3_R) to the dye [117]. This dye can be used for the observation of hH_3_R in HEK-293 cells overexpressing hH_3_R, by fluorescence confocal laser-scanning microscopy, and is used in very small amounts (low concentration: 1−10 nM, hH_3_R-enriched solution: 1 μM). At the same time, it also has a better imaging effect of the inside of the human body, labeled with hH_3_R.

A fluorescein dye has been developed for “naked eye” detection of histamine nanomolar levels in the water. An inexpensive portable ribbon was developed by dropping an MeOH solution of histamine staining agent onto an alumina-coated thin-layer chromatography plate [118]. After air drying, the ribbon showed a strong yellow (green fluorescence under ultraviolet light). Adding histamine-contaminated solution (50 µM) to these thin-layer chromatography plates showed that the color immediately changed from dark yellow to light yellow, and there was a decrease in green fluorescence.

2-(2-Hydroxyphenyl) benzothiazole (HBT) is a fluorophore with a large Stokes shift (180 nm) and an excited-state intramolecular proton transfer (ESIPT) effect. Benefiting from the ESIPT effect, HBT has stable optical properties and a stable AIE effect. The product of acetylation of HBT, HBTAc, can be used for the detection of amine vapor [119]. When the hydroxyl group is acetylated, the ESIPT process is interrupted, so HBTAc does not fluoresce. When HBTAc makes contact with amine, the amine selectively cuts off the acetyl group, thereby restoring the ESIPT effect of HBT, releasing the free hydroxyl group of HBT and generating fluorescence. In this highly selective amine detection process, the AIE effect of HBT can satisfy its solid-state fluorescence emission requirement. Therefore, in further research, HBTAc can be loaded on test strips or hydrogels for amine detection.

Based on the doping of the excellent Histamine Blue in nanoporous silica and surface modification with FC (HB@NPS@FC), a new type of fluorescence sensor was successfully developed [120]. Histamine Blue, based on meso-ionic acid fluoride, reacts specifically with the amine group in histamine through the autocatalytic reaction of the imidazole group in histamine, providing a FRET switching process and a highly differentiated detection of histamine (Figure 4b). This simple HB@NPS@FC sensory platform can accurately determine histamine within the linear response range of 29.12–166.67 µM. The detection limit of the sensor was calculated to be equal to 8.55 µM.

A fluorescence sensor based on AIE to detect and recognize biogenic amines with carboxylic-acid-modified tetraphenylethenes (TPE) was reported [121]. The mixture of carboxylic-acid-modified TPE and biogenic amine exhibited blue emission upon aggregation, which served as an “on” fluorescence sensor for histamine (Figure 4c). The degree of fluorescence enhancement depended on the histamine concentration. The chromium response was used to specifically detect histamine. A fluorescence sensor array showing three TPEs with carboxylic acid groups was able to accurately identify histamine. Additional information on histamine concentration in a “tuna matrix” was also used as an example to verify the test performance of food freshness and quality.

A fluorescent probe (Ni^2+^ and calcein complex) synthesized from calcein and NiCl_2_ was used for histamine measurement and in vivo monitoring [122]. Calcein and NiCl_2_ were used at equal molar concentrations during the synthesis, then mixed in 0.1 M phosphate buffered saline (PBS, pH 7.4). The Ni^2+^ ion acted as a quencher in the probe because the Ni^2+^ ion bound to the iminodiacetic acid moiety can quench the fluorescence of the calcein moiety (Figure 4d). When the fluorescent probe was in contact with histamine, due to the competitive binding, Ni^2+^ ions in the fluorescent probe preferentially combined with histamine to form a histamine-Ni^2+^ complex. Therefore, the fluorescence quenched by Ni^2+^ ions was gradually recovered, and the content of calcein in the solution increased sharply. At the same time, the results of the nuclear magnetic resonance (NMR) spectra also reflected that histamine had a strong affinity for Ni^2+^ ions, which verified the detection process.

#### 2.5.2. Fluorescent Molecular Polymer

The fluorescent dye BODIPY (BDY-hista) was used to label histamine, and a molecularly imprinted polymer was synthesized using a precipitation polymerization method [123]. The polymerization mixture consisted of a template molecule histamine (0.2 mmol), functional monomer methacrylic acid (MAA, 1.6 mmol), crosslinker ethylene glycol dimethacrylate (EDMA, 4 mmol), and initiator 2,2′ Composition-Azobis 2,4-dimethylvaleronitrile (ABDV, 10 mg) in acetonitrile solution, packed in a glass bottle with a sealing septum. It was applied to a similar competitive fluorescence immunoassay.

#### 2.5.3. Organic Solid State Fluorescent Material

Using g-C_3_N_4_ nanosheets as an effective signal probe, a simple new electrochemical luminescent sensor has been reported, which can measure histamine without using any enzymatic reactions or complex modifications [124]. The quenching mechanism is due to the transfer of energy from the excited state of g-C_3_N_4_ to histamine and the inhibition of free radical reactions. The electrochemical luminescence response of the sensor is linearly proportional to the histamine concentration. The range is 0.1 μM to 0.75 mM, and the detection limit is 0.43 nM (R_2_ = 0.98401). It has been successfully applied to the determination of histamine in canned tuna.

A highly fluorescent nanotube composed of asymmetric perylene diimide molecules exhibited high sensitivity (allowing for ppb-level detection) and selectivity for amine vapors [125]. In the fabrication process, a PTFE film was used as the loading material, and the sensor was loaded by dropping the nanotube-containing ethanol suspension (30 μL, 10 μg/mL) onto 1 cm^2^ of the loading material. Meanwhile, the thickness of the nanotube films could be measured by electron microscopy. The fluorescence of nanotube films was quenched upon exposure to amines, due to the photoinduced electron transfer from amines (electron donors) to perylenediimide molecules (electron acceptors) excited in the nanotubes.

The synergy between the anchoring and dilution effect of the cellulose skeleton on the luminous body and the electrostatic repulsion between the aggregation-caused quenching (ACQ) luminous bodies effectively inhibits the aggregation and self-quenching of the luminous body. This general strategy was applied to different ACQ emitters and various partially substituted cellulose derivatives. In addition, the prepared fluorescent solid retains the excellent characteristics of the original cellulose derivatives, such as good solubility and processability. By covalently bonding the ACQ luminophore to the cellulose chain, followed by an ionization process, a series of solid materials with excellent fluorescence emission were realized [126]. Using the active hydroxyl groups on the cellulose chain, we used FITC as an indicator and protoporphyrin IX (PpIX) as an internal reference on cellulose acetate (CA). Subsequently, by simply mixing green-emitting CA-FIT and red-emitting CA-PpIX in different ratios, a cellulose-based ratio fluorescent material with excellent amine response was prepared, which can detect the freshness of seafood in real time and intuitively [127]. They exhibited sensitivity, color response, and a fast and linear response to histamine in a wide range, from 5.0 ppm to 2.5 × 10^4^ ppm.

## 3. Application of Fluorescence Sensors in Detection of Histamine in Fish

It is well known that spoiled fish is the main source of histamine, so the detection of histamine in spoiled fish with a fluorescence sensor can effectively reflect the practical application of the fluorescence sensor. When the fluorescence sensor detects histamine in fish, it faces the influence of complex interfering substances and detection conditions. The influence of detection conditions is mainly reflected in the changes of pH and temperature [128,129]. Therefore, a series of measures is applied to the fluorescence sensor to adapt to the detection of spoiled fish.

In the application of NAC-CQDs, a relatively excellent method for handling fish was demonstrated. Histamine was selectively captured from sample solutions by gold-coated magnetic nanocomposites (MNP@Au) functionalized with cysteine-binding peptides (MNP@Au@peptide), which further separated histamine in fish meat by magnetic field. At the same time, NAC-CQDs had relatively stable fluorescence intensity in the pH range of 6–11, and had a wide pH adaptation range [66].

In the study of EuMOF-FITC, the fluorescence sensor was directly coated on fish samples, and the raw fish were stored at −21 °C, 0 °C, and 25 °C, where the fluorescence of the corresponding films changed significantly at 0 and 25 °C. This process took only one day. This indicated that BAs were quickly detected in large numbers from fish samples at higher temperatures. With increasing exposure time, the color change from orange–red to green was completed within 10 min. EuMOFFITC is sensitive to pH changes because of the covalent linkage between FITC and EuMOF. Since the absorption peak of EuMOF is red-shifted at a high pH, the energy transfer between EuMOF and FITC is more pronounced at this time [81].

During the use of Cy5-A1-949, raw tuna fillets were added with different concentrations of histamine; they were subsequently heated, homogenized, and treated with ultrafiltration to remove macromolecules, while maintaining PH = 7 in the environment [105]. While in MADH, during the use of amicyanin-Cy5, the fish sample was first mixed with 0.1 M sodium phosphate, pH 7.5, and 3 mM sodium azide. It was then homogenized. The homogenate was heated at 90 °C, cooled to room temperature, and finally centrifuged. The supernatant was then ready for detection [112]. In the use of TPEs, extraction from canned tuna with dichloromethane/n-hexane (*v*/*v* 1:1) was utilized [121].

Among these methods are different separations used to detect histamine in fish and examples of sensors directly covering the fish samples being tested. The aptamer selectivity, molecular imprinting, and the material itself overcome the complex environment of histamine detection to some extent. Most sensors specify PH with high fluorescence emission intensity, detect at room temperature, and determine the detection time required for histamine within the time required to detect a stable emission signal.

## 4. Advantages and Challenges of Fluorescence Sensors

We can see from the description of the above fluorescence sensor that for traditional fluorescent dyes, problems such as toxicity, instability, photobleaching, and bio-incompatibility limit their application in the detection of histamine in food. The use of fluorescent nanomaterials can provide a better development environment for fluorescence sensors. Several metal nanoparticles, nanoclusters and upconversion nanoparticles have been used in systems for the fluorescence detection of histamine. Several fluorescence-based metal NPs (e.g., CuNPs, AgNPs and AuNPs) possess high extinction coefficients and size-dependent optical properties. These fluorescence sensors can reflect the histamine content in the environment well, and have high sensitivity (LOD is even less than 1 nM). Among them, AuNPs have attracted the attention of many scholars due to their nontoxicity, biocompatibility, and stability in aqueous solution. However, the aggregation effect that occurs over time also results in the need for expensive stabilizers when used. For UCNPs, although they have good fluorescence properties, they still need to be used in combination with other materials to obtain selective detection capabilities. Cadmium- and zinc-based semiconductor quantum dots have also been used to fabricate histamine fluorescence sensors, which are characterized by narrow fluorescence emission, a broad absorption band, bright fluorescence, and resistance to photobleaching. At the same time, the surface functionalization of these quantum dots can make them show better stability, selectivity, and sensitivity in the process of histamine detection. To overcome the toxicity of some semiconducting quantum dots, CQDs were proposed and applied in the detection of histamine, which exhibited good photoluminescent properties, biocompatibility, and solution stability, and also possessed a low detection limit. Some atom-doped quantum dots have also been proposed to improve quantum yields. GQDs showed strong fluorescence and good biocompatibility during use. At the same time, atomic doping was also used to improve GQDs. In addition, due to their strong fluorescence, good fluorescence stability, functionalization, fast detection rate, and minimal biotoxicity, Pdots can be used as an efficient and good fluorescence sensor for histamine detection, and have considerable optimization potential. For MOFs, their lower solubility and dispersion properties limit their potential for practical applications to a certain extent. At the same time, the introduction of nucleic acid aptamers and molecular imprinting into the fluorescence sensor greatly enhanced its selectivity and sensitivity. Moreover, it also overcomes the complex detection environment caused by the competitive interaction between bioamines with similar structure and sensing materials. However, there is still a lot of research space for the synthesis and customization of aptamers, the linking with fluorescence units, and the mechanism of fluorescence detection.

All in all, an excellent histamine fluorescence sensor needs to have low cost, good fluorescence properties, good water solubility, the ability to avoid biotoxicity, high quantum yield, good sensitivity, and selectivity. The complex detection environment must be considered when applying fluorescence sensors for the detection of histamine in food. At the same time, more and more detailed data and better characterization will facilitate the development of sensors and attract more users, and we have summarized some representative examples in Table 1. In the current research environment, Pdots, combined with aptamers and applied to the detection of histamine, are a good choice.

## 5. Conclusions

This review describes the development of fluorescence sensors for histamine detection in recent decades. From this review, we can see the characteristics of fluorescence sensors: visibility, sensitivity, selectivity, simple operation, and low cost. Considering the complex operating environment for the detection of histamine in food, scholars had made efforts in terms of accuracy and selectivity. Furthermore, high-specificity and high-affinity nucleic acid aptamers and MIPs have been applied to fluorescence sensors to satisfy the detection of histamine in multi-component materials. At the same time, in order to obtain more stable and stronger fluorescence, some sensitive and practical fluorescence sensors have also been reused and improved, such as QDs, MOFs, UCNPs, organic small molecules, etc. In addition, in order to adapt to the needs of production, scholars have also developed a series of fluorescence detection fabrics, fluorescence detection films, and fluorescence sensors based on handheld devices for instant and on-site fluorescence immunoassays. These fluorescent-sensor-based methods for the safe detection of histamine in food have broad potential for research and development. Similar thoughtful method development should help to bring these potentially revolutionary tools to human services, to monitor and ensure food quality, safety, environmental friendliness, and utility.

## Figures and Tables

**Table 1:** Summary of representative examples of fluorescence sensors for the detection of histamine in food.

Type	Name of Fluorescence Sensor (Formula)	LOD	Sample	Excitation/Emission	Linear Range	Size	References
Quantum dots	MP-CNPs	1.5 nM	Fresh plasma	345 nm/410 nm	6.2 nM~0.17 μM	18.6 nm	[49]
	QD-HIOGNF	0.9 nM	Histamine solutions	330 nm/660 nm	0.9~6.3 nM	~3.4 nm	[50]
	QDs@IL@MIP	0.1 mM	Fish	400 nm/605 nm	0.4~2.2 mM	Not available	[53]
	S-doped CD-embedded COF	0.8 nM	Wine and fermented meat products	350 nm/440 nm	1.6~160 nM	5 nm	[54]
	N-CD@Ag^+^	500 nM	Serum	370 nm/440 nm	0.5~100 μM	4~6 nm	[56]
	GQDs-amino	0.056 µM	Tuna fish	345 nm/435 nm	0.4 µM~32µM	28 nm	[57]
	ZnO@PLP/ZnO@Py	0.59 μM/0.97 μM	Analytical grade chemicals Histamine	351 nm/375~700 nm	2.49~24.4 μM/7.44~47.6 μM	3.64 nm	[58]
	PLP_β-CD-AgNPs	0.513 μM	Water	360 nm/515 nm	9.9~31.6 μM	~7 nm	[59]
	TGA-CdTe QDs	160 nM	Fish	350 nm/515 nm	9.8nM~0.6 µM	28.2 nm	[61]
	CdSe Quantum dots/MSA	1.6 µM	White wine	350 nm/380~700 nm	0.07~4.5 mM	2~3 nm	[63]
	NAC-CQDs	13.0 ppb	Blackfish	360 nm/450 nm	0.1~100 ppm	3.3 nm	[66]
	CD-modifified nanoporous alumina membrane	70 pM	Mackerel fish	340 nm/450 nm	0.1 nM~1 mM	10 nm	[67]
Metallic material	EuMOF-FITC	19 µM	Raw fish	380 nm/611nm	45~450 µM	Not available	[81]
	PCL-Por (Zn)	10 µM	Histamine solution	420 nm/700 nm	10~200 µM	455 ± 42 nm	[85]
	Zinc (II) salphen complexes	4.4 pM	Shrimp	300 nm/340–450 nm	10 pM~1 µM	90~160 nm	[86]
	Ag^+4^[PTC]^4–^IOH-NPs	10 µM	Histamine solutions	492 nm/520 nm	Not available	140 ± 8 nm	[90]
	Cu NCs	60 nM	Fish, shrimp and red wine	325 nm/590 nm	0.1~10 µM	<2 nm	[91]
	DPA-CuNPs	30 nM	Fish, pork and red wine	280 nm/650 nm	0.05~5 μM	<0.5 μm	[93]
	UCNPs@MIPs–AgNPs	81 nM	Red wine, rice wine and canned tuna	210 nm/541 nm	0.2µM~2.2 mM	100 nm	[94]
	NaYF4Yb-Er-UCNP	90 pM	Pork, bacon, cheese, wine	980 nm/550 nm	0.9 nM~0.9 μM	30.5 nm	[95]
Aptamer	RNP fluorescence sensor	1 μM	Histamine solutions	390 nm/535 nm.	100 μM~1 mM	Not available	[101]
	aptamer AuNP	8 nM	Fish	350 nm/633 nm	19~70 nM	16 ± 3 nm	[102]
	UR-NR266(12)	15 nM	HEK293-SP-FLAG-hH_3_R cells	550 nm/583 nm	Not available	Not available	[104]
	Cy5-A1-949	1 μM	Tuna	650 nm/670 nm	1~1000 μM	Not available	[105]
Organic small molecules and organic polymers	CE-NBD-F	0.021 µM	Lysate of tobacco Mesophyll protoplasts	400 nm/490 nm	0.05~3.0 μM	Not available	[106]
	Fluorescein dyes for Dual-mode detection	21nM	Water, human serum, urine, cells	457 nm/514 nm	0–0.12mM	Not available	[108]
	CMPT	0.13 µM	Soy sauce	325 nm/475 nm	0.2~4 mM	Not available	[109]
	MADH/amicyanin-Cy5	13 nM	Salmon and human blood serum	645 nm/ 665 nm	13 nM~225 M	Not available	[112]
	Anti-His-LNs	10 ppb	Fish	550 nm/585 nm	15~80 ppb	Not available	[116]
	HB@NPs@FC	8.55 µM	Salmon and tuna	340 nm/536 nm	29.1~166.7 µM	Not available	[120]
	TPEs	50 ppm	Canned tuna fish	350 nm/480 nm	0~100 ppm	Not available	[121]
	Complex between Ni^2+^ and calcein	10 nM	RAW264 cells	485 nm/535 nm	0~1000 μM	Not available	[122]
	Competitive fluorescent MIP	0.02 ppm	Fish	485 nm/528 nm	1~430 µM	278 ± 36 nm	[123]
	Asymmetric PDI molecules	1 ppb	Meat	455 nm/675 nm	Not available	Not available	[125]

## Data Availability

No new data were created or analyzed in this study. Data sharing is not applicable to this article.

## Nomenclature

AOAC	Association of official analytical chemists
QDs	Quantum dots
MIP	Molecularly imprinted polymer
CNPs	Carbon nanoparticles
CDs	Carbon dots
β-CD	β-cyclodextrin
IL	Ionic liquid
COFs	Covalent organic frameworks
GQDs	Graphene quantum dots
MOFs	Metal–organic frameworks
PLP	Pyridoxal phosphate
Py	Pyridoxal
MSA	Mercaptosuccinic acid
IOH-NPs	Inorganic–organic hybrid nanoparticles
AIE	Aggregation-induced emission
UCNPs	Upconversion particles
UCPL	Upconversion photoluminescence
SELEX	Systematic evolution of ligands by exponential enrichment
EIS	Electrochemical impedance spectroscopy
H_3_R	Histamine H_3_ receptors
MADH	Methylamine dehydrogenase
ESIPT	Excited-state intramolecular proton transfer
TPE	Tetraphenylethenes
ACQ	Aggregation-caused quenching
FITC	Fluorescein isothiocyanate

## References

[1] Erdag D., Merhan O., Yildiz B. (2018). Biochemical and pharmacological properties of biogenic amines. Biogenic Amines.

[2] Ladero V., Calles-Enríquez M., Fernández M., Alvarez M.A. (2010). Toxicological effects of dietary biogenic amines. Curr. Nutr. Food Sci..

[3] Kolenbrander P. (2006). The genus Veillonella. Prokaryotes.

[4] Khalid K. (2011). An overview of lactic acid bacteria. Int. J. Biosci..

[5] Yang S.Y., Lin Q., Lu Z.X., Lü F.X., Bie X.M., Zou X.K., Sun L.J. (2008). Characterization of a novel glutamate decarboxylase from *Streptococcus salivarius* ssp. *thermophilus* Y_2_. J. Chem. Technol. Biotechnol. Int. Res. Process Environ. Clean Technol..

[6] Fernández-No I.C., Böhme K., Gallardo J.M., Barros-Velázquez J., Cañas B., Calo-Mata P. (2010). Differential characterization of biogenic amine-producing bacteria involved in food poisoning using MALDI-TOF mass fingerprinting. Electrophoresis.

[7] Emborg J., Laursen B.G., Dalgaard P. (2005). Significant histamine formation in tuna (*Thunnus albacares*) at 2 C—effect of vacuum-and modified atmosphere-packaging on psychrotolerant bacteria. Int. J. Food Microbiol..

[8] Visciano P., Schirone M., Tofalo R., Suzzi G. (2012). Biogenic amines in raw and processed seafood. Front. Microbiol..

[9] Min J., Lee S., Jang A., Jo C., Park C., Lee M. (2007). Relationship between the concentration of biogenic amines and volatile basic nitrogen in fresh beef, pork, and chicken meat. Asian-Australas. J. Anim. Sci..

[10] Suzzi G., Gardini F. (2003). Biogenic amines in dry fermented sausages: A review. Int. J. Food Microbiol..

[11] Lu S., Jiang C., Xu X., Xu C., Li K., Shu R. (2015). Improved screening procedure for biogenic amine production by lactic acid bacteria and Enterobacteria. Czech J. Food Sci..

[12] Demoncheaux J.-P., Michel R., Mazenot C., Duflos G., Iacini C., Delaval F., Saware E., Renard J.-C. (2012). A large outbreak of scombroid fish poisoning associated with eating yellowfin tuna (*Thunnus albacares*) at a military mass catering in Dakar, Senegal. Epidemiol. Infect..

[13] Schirone M., Visciano P., Tofalo R., Suzzi G. (2016). Histamine food poisoning. Histamine and Histamine Receptors in Health and Disease.

[14] Skidgel R.A., Brunton L.L., Hilal-Dandan R., Knollmann B.C. (1996). Histamine, Bradykinin, and Their Antagonists.

[15] Katzung B.G., Julius D. (2000). Histamine, serotonin, and the ergot alkaloids. Basic and Clinical Pharmacology.

[16] Black E., Strada S., Thompson W. (1988). Relationships of secretagogue-induced cAMP accumulation and acid secretion in elutriated rat gastric parietal cells. J. Pharmacol. Methods.

[17] Durak-Dados A., Michalski M., Osek J. (2020). Histamine and other biogenic amines in food. J. Vet. Res..

[18] Spano G., Russo P., Lonvaud-Funel A., Lucas P., Alexandre H., Grandvalet C., Coton E., Coton M., Barnavon L., Bach B. (2010). Biogenic amines in fermented foods. Eur. J. Clin. Nutr..

[19] Ganapathy B., Khalid Q., Ru X. (2017). Histamine content in various types of canned foods (fruits and syrups) stored under different temperature conditions over time-an in vitro study. J. Adv. Biomed. Pathobiol. Res..

[20] Horwitz W., Horwitz W. (1997). Official methods of analysis of AOAC International. Agricultural Chemicals, Contaminants, Drugs.

[21] Jutel M., Akdis M., Akdis C. (2009). Histamine, histamine receptors and their role in immune pathology. Clin. Exp. Allergy.

[22] Dy M., Schneider E. (2004). Histamine–cytokine connection in immunity and hematopoiesis. Cytokine Growth Factor Rev..

[23] Ohtsu H. (2012). Pathophysiologic role of histamine: Evidence clarified by histidine decarboxylase gene knockout mice. Int. Arch. Allergy Immunol..

[24] Feng C., Teuber S., Gershwin M.E. (2016). Histamine (scombroid) fish poisoning: A comprehensive review. Clin. Rev. Allergy Immunol..

[25] Yesudhason P., Al-Zidjali M., Al-Zidjali A., Al-Busaidi M., Al-Waili A., Al-Mazrooei N., Al-Habsi S. (2013). Histamine levels in commercially important fresh and processed fish of Oman with reference to international standards. Food Chem..

[26] Kovacova-Hanuskova E., Buday T., Gavliakova S., Plevkova J. (2015). Histamine, histamine intoxication and intolerance. Allergol. Immunopathol..

[27] Smolinska S., Jutel M., Crameri R., O'mahony L. (2014). Histamine and gut mucosal immune regulation. Allergy.

[28] Henderson P. (1830). Case of poisoning from the bonito (Scomber pelamis). Edinb. Med. J..

[29] Yu Y., Wang P., Bian L., Hong S. (2018). Rare death via histamine poisoning following crab consumption: A case report. J. Forensic Sci..

[30] Köse S., Hall G. (2000). Modification of a colorimetric method for histamine analysis in fish meal. Food Res. Int..

[31] Patange S., Mukundan M., Kumar K.A. (2005). A simple and rapid method for colorimetric determination of histamine in fish flesh. Food Control..

[32] Oguri S., Mizusawa A., Kamada M., Kohori M. (2006). A new method of histamine colorimetry using 2, 3-naphthalenedicarboxaldehyde on a silica–gel column cartridge. Anal. Chim. Acta.

[33] Bi J., Tian C., Zhang G.-L., Hao H., Hou H.-M. (2020). Detection of histamine based on gold nanoparticles with dual sensor system of colorimetric and fluorescence. Foods.

[34] Munzi G., Failla S. (2021). Highly selective and sensitive colorimetric/fluorometric dual mode detection of relevant biogenic amines. Analyst.

[35] Tan A., Zhao Y., Sivashanmugan K., Squire K., Wang A.X. (2019). Quantitative TLC-SERS detection of histamine in seafood with support vector machine analysis. Food Control..

[36] Tahmouzi S., Khaksar R., Ghasemlou M. (2011). Development and validation of an HPLC-FLD method for rapid determination of histamine in skipjack tuna fish (*Katsuwonus pelamis*). Food Chem..

[37] Cicero A., Galluzzo F.G., Cammilleri G., Pulvirenti A., Giangrosso G., Macaluso A., Vella A., Ferrantelli V. (2020). Development of a rapid and eco-friendly UHPLC analytical method for the detection of histamine in fish products. Int. J. Environ. Res. Public Health.

[38] Hwang B.-S., Wang J.-T., Choong Y.-M. (2003). A rapid gas chromatographic method for the determination of histamine in fish and fish products. Food Chem..

[39] Klimov V., Mikhailovsky A., Xu S., Malko A., Hollingsworth J., Leatherdale A.C., Eisler H.-J., Bawendi M. (2000). Optical gain and stimulated emission in nanocrystal quantum dots. Science.

[40] Yu W.W., Peng X. (2007). Formation of High-Quality CdS and Other II–VI Semiconductor Nanocrystals in Noncoordinating Solvents: Tunable Reactivity of Monomers. Angew. Chem..

[41] Nirmal M., Brus L. (1999). Luminescence photophysics in semiconductor nanocrystals. Acc. Chem. Res..

[42] Li M., Chen T., Gooding J.J., Liu J. (2019). Review of carbon and graphene quantum dots for sensing. ACS Sens..

[43] Molaei M.J. (2020). Principles, mechanisms, and application of carbon quantum dots in sensors: A review. Anal. Methods.

[44] Duan J., Jiang X., Ni S., Yang M., Zhan J. (2011). Facile synthesis of N-acetyl-l-cysteine capped ZnS quantum dots as an eco-friendly fluorescence sensor for Hg^2+^. Talanta.

[45] Zhang Y., Xiao J.-Y., Zhu Y., Tian L.-J., Wang W.-K., Zhu T.-T., Li W.-W., Yu H.-Q. (2020). Fluorescence sensor based on biosynthetic CdSe/CdS quantum dots and liposome carrier signal amplification for mercury detection. Anal. Chem..

[46] Nan Z., Hao C., Zhang X., Liu H., Sun R. (2020). Carbon quantum dots (CQDs) modified ZnO/CdS nanoparticles based fluorescence sensor for highly selective and sensitive detection of Fe (III). Spectrochim. Acta Part A Mol. Biomol. Spectrosc..

[47] Liang J., Yu L., Li X., Zhang J., Chen G., Zhang J. (2019). Self-assembled quantum dot microstructure guided by a microemulsion approach for immunoassays. RSC Adv..

[48] Costa W.C., Salla C.A., Ely F., Bechtold I.H. (2021). Highly emissive MAPbBr_3_ perovskite QDs by ligand-assisted reprecipitation: The antisolvent effect. Nanotechnology.

[49] Zakery M., Ensafi A.A., Kazemifard N., Rezaei B. (2020). Novel Histamine Fluorosensor Based on Modified Environmental Friendly Carbon Nanoparticles From Gum Tragacanth. IEEE Sens. J..

[50] Kim Y., Chang J.Y. (2016). Fabrication of a fluorescent sensor by organogelation: CdSe/ZnS quantum dots embedded molecularly imprinted organogel nanofibers. Sens. Actuators B Chem..

[51] Johnson K.E. (2007). What’s an ionic liquid?. Electrochem. Soc. Interface.

[52] Wilkes J.S. (2004). Properties of ionic liquid solvents for catalysis. J. Mol. Catal. A: Chem..

[53] Wang Q.-H., Fang G.-Z., Liu Y.-Y., Zhang D.-D., Liu J.-M., Wang S. (2017). Fluorescent sensing probe for the sensitive detection of histamine based on molecular imprinting ionic liquid-modified quantum dots. Food Anal. Methods.

[54] Zhang D., Wang Y., Xie J., Geng W., Liu H. (2020). Ionic-liquid-stabilized fluorescent probe based on S-doped carbon dot-embedded covalent-organic frameworks for determination of histamine. Microchim. Acta.

[55] Yuan X., Liu H., Sun B. (2020). N-doped carbon dots derived from covalent organic frameworks embedded in molecularly imprinted polymers for optosensing of flonicamid. Microchem. J..

[56] Du N., Dou Z., Wu Y., Wu Q., Zhang G., Liu X. (2020). Fluorescent nanoprobe array based on carbon nanodots for qualitative and quantitative determination of biogenic polyamine. Microchim. Acta.

[57] Toloza C.A., Khan S., Silva R.L., Romani E.C., Larrude D.G., Louro S.R., Júnior F.L.F., Aucelio R.Q. (2017). Photoluminescence suppression effect caused by histamine on amino-functionalized graphene quantum dots with the mediation of Fe^3+^, Cu^2+^, Eu^3+^: Application in the analysis of spoiled tuna fish. Microchem. J..

[58] Yadav A., Upadhyay Y., Bera R.K., Sahoo S.K. (2020). Vitamin B6 cofactors guided highly selective fluorescent turn-on sensing of histamine using beta-cyclodextrin stabilized ZnO quantum dots. Food Chem..

[59] Bhardwaj V., Kumar S.A., Sahoo S.K. (2020). Decorating Vitamin B6 Cofactor over Beta-Cyclodextrin Stabilized Silver Nanoparticles through Inclusion Complexation for Fluorescent Turn-On Detection of Hydrazine. ACS Appl. Bio Mater..

[60] Viswanath A., Shen Y., Green A.N., Tan R., Greytak A.B., Benicewicz B.C. (2014). Copolymerization and synthesis of multiply binding histamine ligands for the robust functionalization of quantum dots. Macromolecules.

[61] Khan S., Carneiro L.S., Vianna M.S., Romani E.C., Aucelio R.Q. (2017). Determination of histamine in tuna fish by photoluminescence sensing using thioglycolic acid modified CdTe quantum dots and cationic solid phase extraction. J. Lumin..

[62] Li L., Qian H., Fang N., Ren J. (2006). Significant enhancement of the quantum yield of CdTe nanocrystals synthesized in aqueous phase by controlling the pH and concentrations of precursor solutions. J. Lumin..

[63] Gagic M., Nejdl L., Xhaxhiu K., Cernei N., Zitka O., Jamroz E., Svec P., Richtera L., Kopel P., Milosavljevic V. (2020). Fully automated process for histamine detection based on magnetic separation and fluorescence detection. Talanta.

[64] Sarikaya M., Tamerler C., Jen A.K.-Y., Schulten K., Baneyx F. (2003). Molecular biomimetics: Nanotechnology through biology. Nat. Mater..

[65] Azzazy H.M., Highsmith W.E. (2002). Phage display technology: Clinical applications and recent innovations. Clin. Biochem..

[66] Shi R., Feng S., Park C.Y., Park K.Y., Song J., Park J.P., Chun H.S., Park T.J. (2020). Fluorescence detection of histamine based on specific binding bioreceptors and carbon quantum dots. Biosens. Bioelectron..

[67] Mao Y., Zhang Y., Hu W., Ye W. (2019). Carbon dots-modified nanoporous membrane and Fe_3_O_4_@ Au magnet nanocomposites-based FRET assay for ultrasensitive histamine detection. Molecules.

[68] Hu W., Chen T., Zhang Y., Ye W. (2019). A carbon dot and gold nanoparticle-based fluorometric immunoassay for 8-hydroxy-2′-deoxyguanosine in oxidatively damaged DNA. Microchim. Acta.

[69] Shi Y., Lin L., Wei Y., Li W., Nie P., He Y., Feng X. (2021). Gold nanoparticles-mediated ratiometric fluorescence aptasensor for ultra-sensitive detection of Abscisic Acid. Biosens. Bioelectron..

[70] Lin J., Su Q. (1995). Luminescence and energy transfer of rare-earth-metal ions in Mg_2_Y_8_ (SiO_4_)_6_O_2_. J. Mater. Chem..

[71] Sreeram K.J., Aby C.P., Nair B.U., Ramasami T. (2008). Colored cool colorants based on rare earth metal ions. Sol. Energy Mater. Sol. Cells.

[72] Chia Y., Tay M. (2014). An insight into fluorescent transition metal complexes. Dalton Trans..

[73] Diamandis E.P. (1988). Immunoassays with time-resolved fluorescence spectroscopy: Principles and applications. Clin. Biochem..

[74] Dou X., Sun K., Chen H., Jiang Y., Wu L., Mei J., Ding Z., Xie J. (2021). Nanoscale metal-organic frameworks as fluorescence sensors for food safety. Antibiotics.

[75] Cui Y., Song R., Yu J., Liu M., Wang Z., Wu C., Yang Y., Wang Z., Chen B., Qian G. (2015). Dual-emitting MOF⊃ dye composite for ratiometric temperature sensing. Adv. Mater..

[76] Yin H.-Q., Wang X.-Y., Yin X.-B. (2019). Rotation restricted emission and antenna effect in single metal–organic frameworks. J. Am. Chem. Soc..

[77] Yin H.-Q., Yin X.-B. (2020). Metal–organic frameworks with multiple luminescence emissions: Designs and applications. Acc. Chem. Res..

[78] Xu X.-Y., Yan B. (2017). Eu (III)-functionalized ZnO@ MOF heterostructures: Integration of pre-concentration and efficient charge transfer for the fabrication of a ppb-level sensing platform for volatile aldehyde gases in vehicles. J. Mater. Chem. A.

[79] Zhang W.-Q., Li Q.-Y., Cheng J.-Y., Cheng K., Yang X., Li Y., Zhao X., Wang X.-J. (2017). Ratiometric Luminescent Detection of Organic Amines Due to the Induced Lactam–Lactim Tautomerization of Organic Linker in a Metal–Organic Framework. ACS Appl. Mater. Interfaces.

[80] Ni J., Li M.-Y., Liu Z., Zhao H., Zhang J.-J., Liu S.-Q., Chen J., Duan C.-Y., Chen L.-Y., Song X.-D. (2020). Discrimination of various amine vapors by a triemissive metal-organic framework composite via the combination of a three-dimensional ratiometric approach and a confinement-induced enhancement effect. ACS Appl. Mater. Interfaces.

[81] Wang J., Li D., Ye Y., Qiu Y., Liu J., Huang L., Liang B., Chen B. (2021). A Fluorescent Metal-Organic Framework for Food Real-Time Visual Monitoring. Adv. Mater..

[82] Li P., Guo M.-Y., Gao L.-L., Yin X.-M., Yang S.-L., Bu R., Gao E.-Q. (2020). Photoresponsivity and antibiotic sensing properties of an entangled tris (pyridinium)-based metal–organic framework. Dalton Trans..

[83] Xu X.Y., Lian X., Hao J.N., Zhang C., Yan B. (2017). A double-stimuli-responsive fluorescent center for monitoring of food spoilage based on dye covalently modified EuMOFs: From sensory hydrogels to logic devices. Adv. Mater..

[84] Recillas-Mota J., Bernad-Bernad M., Gracia-Mora J. (2009). Histamine interaction with Zn^2+^ and Cu^2+^ porphyrins. Die Pharm. -Int. J. Pharm. Sci..

[85] Choi M.-S., Kim Y.-J. (2012). Fluorescent chemosensor for the detection of histamine based on dendritic porphyrin-incorporated nanofibers. Eur. Polym. J..

[86] Sahudin M.A., Su’ait M.S., Tan L.L., Lee Y.H., Abd Karim N.H. (2019). Zinc (II) salphen complex-based fluorescence optical sensor for biogenic amine detection. Anal. Bioanal. Chem..

[87] Guo Q.-N., Li Z.-Y., Chan W.-H., Lau K.-C., Crossley M.J. (2010). Appending zinc tetraphenylporphyrin with an amine receptor at β-pyrrolic carbon for designing a selective histamine chemosensor. Supramol. Chem..

[88] Oshikawa Y., Furuta K., Tanaka S., Ojida A. (2016). Cell surface-anchored fluorescent probe capable of real-time imaging of single mast cell degranulation based on histamine-induced coordination displacement. Anal. Chem..

[89] Neumeier B.L., Khorenko M., Alves F., Goldmann O., Napp J., Schepers U., Reichardt H.M., Feldmann C. (2019). Fluorescent Inorganic-Organic Hybrid Nanoparticles. ChemNanoMat.

[90] Neumeier B.L., Heck J.G., Feldmann C. (2019). Fluorescence-based histamine sensing with inorganic–organic hybrid nanoparticles. J. Mater. Chem. C.

[91] Han A., Xiong L., Hao S., Yang Y., Li X., Fang G., Liu J., Pei Y., Wang S. (2018). Highly bright self-assembled copper nanoclusters: A novel photoluminescent probe for sensitive detection of histamine. Anal. Chem..

[92] Chen Z., Ding Z., Zhang G., Tian L., Zhang X. (2018). Construction of thermo-responsive elastin-like polypeptides (ELPs)-aggregation-induced-emission (AIE) conjugates for temperature sensing. Molecules.

[93] Zhang X., Liu Q., Wang Z.-W., Xu H., An F.-P., Huang Q., Song H.-B., Wang Y.-W. (2020). D-penicillamine modified copper nanoparticles for fluorometric determination of histamine based on aggregation-induced emission. Microchim. Acta.

[94] Wu Z., Xu E., Jiao A., Jin Z., Irudayaraj J. (2017). Bimodal counterpropagating-responsive sensing material for the detection of histamine. Rsc Adv..

[95] Zhang B., Sheng W., Liu Y., Huang N., Zhang W., Wang S. (2020). Multiplexed fluorescence immunoassay combined with magnetic separation using upconversion nanoparticles as multicolor labels for the simultaneous detection of tyramine and histamine in food samples. Anal. Chim. Acta.

[96] Deng T., Yan S., Jiang X., Zhang Q. (2019). Improving Upconversion Photoluminescence of GdAl [O. sub.^3+^]:[Er. sup.^3+^], [Yb. sup.^3+^] Phosphors via [Ga. sup.^3+^], [Lu. sup.^3+^] Doping. Int. J. Opt..

[97] Siegel P.D., Lewis D.M., Petersen M., Olenchock S.A. (1990). Observations on the use of o-phthalaldehyde condensation for the measurement of histamine. Analyst.

[98] Tsikas D., Velásquez R., Toledano C., Brunner G. (1993). Ion-pair extraction of histamine from biological fluids and tissues for its determination by high-performance liquid chromatography with fluorescence detection. J. Chromatogr. B Biomed. Sci. Appl..

[99] Mairal Lerga T., Jauset-Rubio M., Skouridou V., Bashammakh A.S., El-Shahawi M.S., Alyoubi A.O., O’Sullivan C.K. (2019). High affinity aptamer for the detection of the biogenic amine histamine. Anal. Chem..

[100] Ho L.S.J., Fogel R., Limson J.L. (2020). Generation and screening of histamine-specific aptamers for application in a novel impedimetric aptamer-based sensor. Talanta.

[101] Liew F.F., Fukuda M., Morii T. (2010). Development of Fluorescent Ribonucleopeptide-Based Sensors for Biologically Active Amines. Zero-Carbon Energy Kyoto 2009.

[102] Lerga T.M., Skouridou V., Bermudo M.C., Bashammakh A.S., El-Shahawi M.S., Alyoubi A.O., O’Sullivan C.K. (2020). Gold nanoparticle aptamer assay for the determination of histamine in foodstuffs. Microchim. Acta.

[103] Bartole E., Grätz L., Littmann T., Wifling D., Seibel U., Buschauer A., Bernhardt G.n. (2020). UR-DEBa242: A Py-5-Labeled fluorescent multipurpose probe for investigations on the histamine H_3_ and H_4_ receptors. J. Med. Chem..

[104] Rosier N., Grätz L., Schihada H., Möller J., Işbilir A., Humphrys L.J., Nagl M., Seibel U., Lohse M.J., Pockes S. (2021). A Versatile Sub-Nanomolar Fluorescent Ligand Enables NanoBRET Binding Studies and Single-Molecule Microscopy at the Histamine H3 Receptor. J. Med. Chem..

[105] Dwidar M., Yokobayashi Y. (2019). Development of a histamine aptasensor for food safety monitoring. Sci. Rep..

[106] Zhang L.-Y., Tang X.-C., Sun M.-X. (2005). Simultaneous determination of histamine and polyamines by capillary zone electrophoresis with 4-fluor-7-nitro-2, 1, 3-benzoxadiazole derivatization and fluorescence detection. J. Chromatogr. B.

[107] Ali A., Kamra M., Roy S., Muniyappa K., Bhattacharya S. (2017). Enhanced G-quadruplex DNA stabilization and telomerase inhibition by novel fluorescein derived salen and salphen based Ni (II) and Pd (II) complexes. Bioconjugate Chem..

[108] Dey N., Ali A., Podder S., Majumdar S., Nandi D., Bhattacharya S. (2017). Dual-Mode Optical Sensing of Histamine at Nanomolar Concentrations in Complex Biological Fluids and Living Cells. Chem. Eur. J..

[109] Nakano T., Todoroki K., Ishii Y., Miyauchi C., Palee A., Min J.Z., Inoue K., Suzuki K., Toyo’oka T. (2015). An easy-to-use excimer fluorescence derivatization reagent, 2-chloro-4-methoxy-6-(4-(pyren-4-yl) butoxy)-1, 3, 5-triazine, for use in the highly sensitive and selective liquid chromatography analysis of histamine in Japanese soy sauces. Anal. Chim. Acta.

[110] Seto D., Soh N., Nakano K., Imato T. (2010). An amphiphilic fluorescent probe for the visualization of histamine in living cells. Bioorganic Med. Chem. Lett..

[111] Ding Z., Dou X., Wang C., Feng G., Xie J., Zhang X. (2021). Ratiometric pH sensing by fluorescence resonance energy transfer-based hybrid semiconducting polymer dots in living cells. Nanotechnology.

[112] Gustiananda M., Andreoni A., Tabares L.C., Tepper A.W., Fortunato L., Aartsma T.J., Canters G.W. (2012). Sensitive detection of histamine using fluorescently labeled oxido-reductases. Biosens. Bioelectron..

[113] Ou L.-J., Liu S.-J., Chu X., Shen G.-L., Yu R.-Q. (2009). DNA encapsulating liposome based rolling circle amplification immunoassay as a versatile platform for ultrasensitive detection of protein. Anal. Chem..

[114] Nie D., Guo D., Huang Q., Guo W., Wang J., Zhao Z., Han Z. (2021). A novel insight into fluorescent sensor for patulin detection using thiol-terminated liposomes with encapsulated coumarin-6 as signal probe. Sens. Actuators B Chem..

[115] Zhao X., Shippy S.A. (2004). Competitive immunoassay for microliter protein samples with magnetic beads and near-infrared fluorescence detection. Anal. Chem..

[116] Bajpai V.K., Oh C., Khan I., Haldorai Y., Gandhi S., Lee H., Song X., Kim M., Upadhyay A., Chen L. (2020). Fluorescent immunoliposomal nanovesicles for rapid multi-well immuno-biosensing of histamine in fish samples. Chemosphere.

[117] Tomasch M., Schwed J.S., Paulke A., Stark H. (2013). Bodilisant A Novel Fluorescent, Highly Affine Histamine H_3_ Receptor Ligand. ACS Med. Chem. Lett..

[118] Wang Q., Barge L.M., Steinbock O. (2019). Microfluidic production of pyrophosphate catalyzed by mineral membranes with steep pH gradients. Chem. Eur. J..

[119] Bao C., Shao S., Zhou H., Han Y. (2021). A new ESIPT-based fluorescent probe for the highly sensitive detection of amine vapors. New J. Chem..

[120] Chaicham A., Kongwutthivech J., Tuntulani T., Tomapatanaget B. (2018). Couple of Histamine blue fluorescence chemosensor and surface charge selector of FC-modified silica nanoporous for highly specific histamine detection via FRET-process. Sens. Actuators B Chem..

[121] Nakamura M., Sanji T., Tanaka M. (2011). Fluorometric sensing of biogenic amines with aggregation-induced emission-active tetraphenylethenes. Chem. Eur. J..

[122] Seto D., Soh N., Nakano K., Imato T. (2010). Selective fluorescence detection of histamine based on ligand exchange mechanism and its application to biomonitoring. Anal. Biochem..

[123] Mattsson L., Xu J., Preininger C., Bui B.T.S., Haupt K. (2018). Competitive fluorescent pseudo-immunoassay exploiting molecularly imprinted polymers for the detection of biogenic amines in fish matrix. Talanta.

[124] Mesgari F., Salehnia F., Beigi S.M., Hosseini M., Ganjali M.R. (2021). Enzyme Free Electrochemiluminescence Sensor of Histamine Based on Graphite-carbon Nitride Nanosheets. Electroanalysis.

[125] Hu Y., Ma X., Zhang Y., Che Y., Zhao J. (2016). Detection of amines with fluorescent nanotubes: Applications in the assessment of meat spoilage. Acs Sens..

[126] Tian W., Zhang J., Yu J., Wu J., Nawaz H., Zhang J., He J., Wang F. (2016). Cellulose-based solid fluorescent materials. Adv. Opt. Mater..

[127] Jia R., Tian W., Bai H., Zhang J., Wang S., Zhang J. (2019). Amine-responsive cellulose-based ratiometric fluorescent materials for real-time and visual detection of shrimp and crab freshness. Nat. Commun..

[128] Ding Z., Wang C., Feng G., Zhang X. (2018). Thermo-responsive fluorescent polymers with diverse LCSTs for ratiometric temperature sensing through FRET. Polymers.

[129] Ding Z., Wang C., Wang S., Wu L., Zhang X. (2019). Light-harvesting metal-organic framework nanoprobes for ratiometric fluorescence energy transfer-based determination of pH values and temperature. Microchim. Acta.

